# Moyamoya Syndrome Secondary to Coinfection of Meningovascular Neurosyphilis and Acquired Immunodeficiency Syndrome

**DOI:** 10.7759/cureus.60579

**Published:** 2024-05-19

**Authors:** Gursan G Uygun, Didem Darici, Melis G Cil, Zeynep Bastug, Cemile H Misirli

**Affiliations:** 1 Department of Neurology, University of Health Sciences, Haydarpasa Numune Training and Research Hospital, Istanbul, TUR; 2 Department of Interventional Neurology, University of Health Sciences, Sancaktepe Sehit Prof. Dr. Ilhan Varank Training and Research Hospital, Istanbul, TUR

**Keywords:** moyamoya etiology, neurosyphilis, human immunodeficiency virus (hiv) infection, young onset stroke, moyamoya angiopathy

## Abstract

Moyamoya angiopathy is a rare cerebrovascular condition characterized by insufficient cerebral blood flow resulting from arterial vessel narrowing or occlusion, potentially leading to cerebral ischemia due to inadequate oxygen and nutrient supply to the brain tissue. The development of collateral vessels in stenotic regions, inherently fragile and prone to rupture, may further precipitate intracerebral hemorrhage. Alongside focal neurological symptoms, the common clinical presentations of Moyamoya angiopathy encompass headaches, dizziness, cognitive impairments, seizures, and involuntary movements. When associated with an underlying systemic illness, including Down Syndrome, cranial radiation, neurofibromatosis type 1, or meningitis, the condition is termed Moyamoya syndrome; whereas when idiopathic and a genetic mutation are identified, it is referred to as Moyamoya disease. In this report, we present a case of the rare Moyamoya syndrome, which was attributed to syphilis and HIV infection and was identified during an investigation into the etiology of ischemic stroke in a young patient.

## Introduction

Moyamoya angiopathy is characterized by the progressive narrowing of the terminal part of the internal carotid arteries, the proximal parts of the middle and anterior cerebral arteries, and a collateral vascular network near these narrowings, which gives rise to a "smoke-like" appearance known as Moyamoya vessels [1]. Exhibited disorders such as vascular morphological appearance on angiographic imaging are classified as Moyamoya angiopathy. When idiopathic and in the presence of a genetic mutation, it is defined as Moyamoya disease; when associated with a condition or disease, it is termed Moyamoya syndrome (MMS) [2]. MMS caused by meningitis is rare, constituting approximately 2.2% of all disorders classified as MMS, where delayed-onset morphological changes in vascular structure may be observed. Moreover, elevated levels of autoantibodies could suggest an autoimmune stimulus for the initiation of MMS [3].

## Case presentation

**Figure 1:**
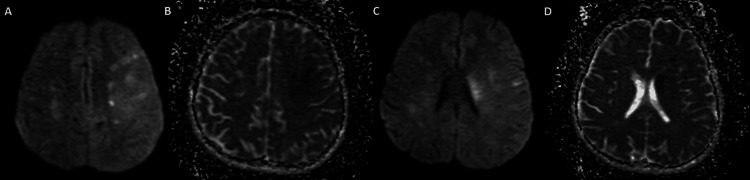
Multiple left cerebral internal border zone infarcts demonstrating restricted diffusion These appear hyperintense on DWI (A, C) with corresponding ADC images (B, D) showing dark signals. DWI: diffusion-weighted imaging; ADC: apparent diffusion coefficient

A 26-year-old male patient was brought to the emergency department due to sudden right-sided weakness and inability to speak. Neurological examination revealed global aphasia and muscle weakness in the right upper and lower extremities graded 3/5. Computed tomography angiography (CTA) of the neck and brain showed a filling defect in the proximal segment of the left middle cerebral artery (MCA), and diffusion-weighted magnetic resonance imaging (MRI) revealed acute ischemic infarcts in the left centrum semiovale with an anterior-posterior distribution (Figure 1).

**Figure 2:**
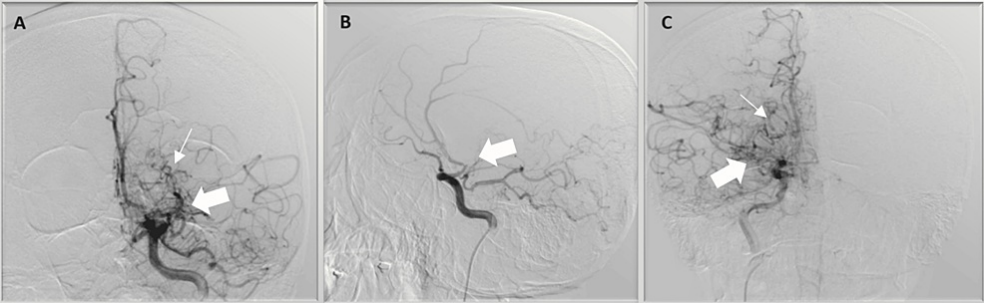
In the left internal carotid artery injection, (A) anterior-posterior and (B) lateral projection, and in the right internal carotid artery injection, (C) anterior-posterior projection shows stenosis with thick arrows, collateral vessels causing a "smoke-like" appearance with thin arrows

Intravenous thrombolytic therapy was initiated three hours after the onset of symptoms and the patient was taken to the angiography suite for a mechanical thrombectomy. Digital subtraction angiography (DSA) demonstrated severe stenosis of the bilateral internal carotid arteries (ICA) at the supraclinoid segment, and increased vascular collateral filling in the striatocapsular area (Figure 2). No thrombus was seen in the lumen, a thrombectomy procedure could not be performed, and it was evaluated as chronic vasculopathy.

The hemogram, biochemistry, electrocardiography, transthoracic echocardiography, and lipid profile included in the assessment of risk factors were normal.

Skin and mucosal examination revealed lesions on the hard palate and palmoplantar area and nodular formation on the penis. In the examination of the lymph system, lymph adenopathy was detected in the post-cervical, right submandibular, left jugular area, and both inguinal regions. The presence of lymph node involvement in more than two regions suggested that LAP was generalized in our patient, and generalized lymph node involvement was thought to be an indicator of a systemic disease. We utilized screening panels for autoimmune tests and infectious pathogens. Anti-HIV was positive, HIV-RNA was 516,121 copies/ml, CD4 count was 160 cells/mm^3^, serum Treponema pallidum haemagglutination assay (TPHA) was positive, venereal disease research laboratory (VDRL) was 159 positive, and rapid plasma reagin (RPR) was considered positive at a dilution of 1/32. In the cerebrospinal fluid (CSF), the cell count was 44/mm^3^, glucose was 38 mg/dl, and protein was 186 mg/dl. The CSF/serum glucose ratio is 0.37. The CSF meningitis panel, tuberculosis, HSV-1/2, and VZV DNA PCR were negative; CSF VDRL and TPHA were found to be positive. The diagnosis of acquired immune deficiency syndrome and neurosyphilis was confirmed.

**Figure 3:**
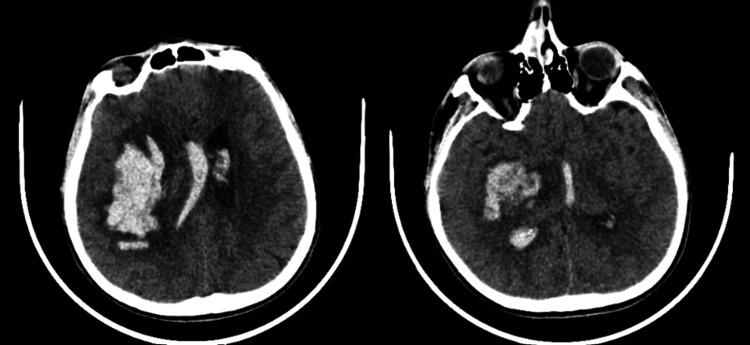
Brain computed tomography imaging showing the hematoma causing a shift of the midline and opening into the ventricular system

Antibiotic therapy and antiretroviral treatment were initiated. On the twentieth day of hospitalization, the patient presented with a fever of 38.4°C, impaired consciousness, and subsequently experienced a seizure. Following the seizure, the patient became hypertensive. A scheduled brain imaging revealed a parenchymal hematoma and intraventricular hemorrhage in the contralateral hemisphere with underlying ischemia (Figure 3). The patient's condition deteriorated, culminating in mortality.

## Discussion

Moyamoya angiopathy, one of the rare causes of ischemic stroke, can be associated with infection. In cases of meningitis associated with pathogens such as Streptococcus pneumoniae, Haemophilus influenzae, and Treponema pallidum, it has been indicated that cerebral vascular structures are affected and can lead to Moyamoya syndrome. It has been mentioned that the infection can trigger an autoimmune process targeting cerebral vascular structures, and this process may play a role in the pathogenesis of infection-associated vasculopathy [4].

In the literature, Moyamoya syndrome has been reported twice in association with the coexistence of HIV and neurosyphilis infection. Morgello and Laufer presented a 22-year-old male patient who had been experiencing headaches, nausea, and subsequent right-sided weakness for 2 months and had undergone lumbar puncture (LP) with a cerebrospinal fluid (CSF) sample showing 98% mononuclear cells, glucose 11 mg/dL, and protein 217 mg/dl, and a negative CSF-VDRL. The patient, who was being treated for tuberculous meningitis, returned eight months later with complaints of blurred vision and forgetfulness, and an ischemic infarct was observed on brain CT. Cerebral angiography showed multiple stenosis and occlusion areas in the branches of the Willis polygon and hypertrophy in the collateral perforating branches. The autopsy of the patient, who died 15 months after the onset of symptoms, showed fibrotic leptomeninges, gummatous arteritis in the major arteries at the base of the brain, intimal and medial proliferation, and lymphocytic infiltration (Heubner's arteritis) in the smaller arteries of the leptomeninges and neural parenchyma. A "beaded appearance" was mentioned in the basilar artery, and HIV was identified in the monocytes taken from the lumen. In this case, a radiological appearance similar to Moyamoya was mentioned for the first time in HIV and neurosyphilis [5].

The second case was presented by Wilson et al., involving a 64-year-old patient who presented with acute right-sided weakness and dysphasia. The patient was initially discharged with aspirin treatment. However, the patient returned with similar symptoms six months later and was diagnosed with a contrast-enhancing mass in the basal ganglia on planned cranial MRI and stenosis in the bilateral ICA terminal and MCA M1 segment on brain CT angiography. In the biopsy of basal ganglia material taken from this patient, lymphocytic and inflammatory cell infiltration and vascular wall were observed, and when the imaging findings were evaluated together, it was considered as probable vasculitis. After the patient had a seizure, a diffusion-weighted MRI taken during the third admission showed a watershed infarct in the left frontoparietal area. Cerebral angiography revealed stenosis in the left ICA supraclinoid segment and collateral networks with a "smoke" characteristic appearance in the left MCA M1 segment. During the examination, skin lesions suggestive of Kaposi's sarcoma were observed. Given the elevated HIV-RNA copy number and positive VDRL in both serum and CSF, the patient was diagnosed with HIV and neurosyphilis infection [6].

We diagnosed Moyamoya angiopathy shortly after admission, prompted by the sudden onset of the patient's symptoms and immediate angiography suite admission for thrombectomy. In the literature, clinical symptoms manifested as ischemic stroke in two cases. In our case, in addition to the ischemic stroke, intracranial hemorrhage occurred in the contralateral hemisphere after a period of time.

Intracranial hematoma occurs in 10% of adult Moyamoya patients [7]. Factors causing hemorrhage in Moyamoya disease include dilation and abnormal branching of the anterior choroidal artery (AChA) and/or posterior communicating artery (PComA). Dilated perforating arteries may rupture due to prolonged hemodynamic stress, and increased pressure on AChA and PComA from ICA distal stenosis could elevate rupture risk [8]. Collateral vessels in periventricular areas may be fragile due to abnormal vascular structures [9].

At the time of admission and throughout the follow-up period, the patient's blood pressure remained within the normal range. However, following the generalized tonic-clonic seizure, there was a fluctuation in blood pressure, becoming hypertensive. Seizures are a frequently observed symptom in Moyamoya syndrome patients and are often associated with disease progression [10]. Elevated fever resulting from immunodeficiency and infection, along with post-seizure hypertension, can increase the risk of hemorrhage. Particularly, collateral vessels inherent to the nature of the disease may be fragile due to abnormal vascular structures, further augmenting the risk of bleeding. The interplay of these factors likely contributed to the occurrence of hemorrhage in the patient.

Early recognition of the underlying condition causing Moyamoya syndrome and prompt initiation of treatment can have a positive impact on the prognosis. The severity of neurological symptoms at the time of diagnosis can also significantly influence the prognosis, with patients experiencing milder symptoms having a better outlook compared to those with more severe symptoms or a history of recurrent strokes. Additionally, epileptic seizures may occur depending on the extent of cerebral hypoperfusion or the presence of infarcts or ischemic lesions, and they can worsen the prognosis [10]. Suzuki's angiographic stage IV or higher is indicative of severe disease [11]. Moreover, intracranial hemorrhage stands as the primary cause of neurological deficits and contributes to a poor prognosis [8].

Our case is the first to report intracranial hematoma occurring in the contralateral hemisphere in addition to ischemic stroke, where HIV and neurosyphilis infection played a role in the etiology of Moyamoya syndrome.

The advancement of our patient's HIV infection to the stage of immunodeficiency, recurrence of fever during antimicrobial therapy, epileptic seizures, ischemic stroke, and intracerebral hemorrhage have significantly worsened the prognosis.

This case presentation indicates the need for careful evaluation of neurological complications and rare conditions such as Moyamoya syndrome in the young adult population with the co-existence of HIV and syphilis infection.

## Conclusions

Moyamoya syndrome can be one of the causes of infectious vasculitis. Investigating the cause and determining the prognosis is crucial. This emphasizes the need for further research, increased knowledge, and a multidisciplinary approach involving the fields of infectious diseases and neurology in the management of such rare conditions.

## References

[1] Scott RM, Smith ER (2009). Moyamoya disease and Moyamoya syndrome. N Engl J Med.

[2] Kuroda S, Fujimura M, Takahashi J (2022). Diagnostic criteria for Moyamoya disease - 2021 revised version. Neurol Med Chir (Tokyo).

[3] Fox BM, Dorschel KB, Lawton MT, Wanebo JE (2021). Pathophysiology of vascular stenosis and remodeling in Moyamoya disease. Front Neurol.

[4] Czartoski T, Hallam D, Lacy JM, Chun MR, Becker K (2005). Postinfectious vasculopathy with evolution to moyamoya syndrome. J Neurol Neurosurg Psychiatry.

[5] Morgello S, Laufer H (1989). Quaternary neurosyphilis in a Haitian man with human immunodeficiency virus infection. Hum Pathol.

[6] Wilson BC, Bear M, Srinivasan A, Rizvi K, Elfallal S, Fang X, Ezzeldin M (2021). Rapidly progressing moyamoya syndrome secondary to meningovascular neurosyphilis and acquired immunodeficiency syndrome. Cureus.

[7] Zafar SF, Bershad EM, Gildersleeve KL, Newmark ME, Calvillo E, Suarez JI, Venkatasubba Rao CP (2014). Adult moyamoya disease in an urban center in the United States is associated with a high burden of watershed ischemia. J Am Heart Assoc.

[8] Morioka M, Hamada J, Kawano T, Todaka T, Yano S, Kai Y, Ushio Y (2003). Angiographic dilatation and branch extension of the anterior choroidal and posterior communicating arteries are predictors of hemorrhage in adult moyamoya patients. Stroke.

[9] Yamashita M, Oka K, Tanaka K (1983). Histopathology of the brain vascular network in moyamoya disease. Stroke.

[10] Mikami T, Ochi S, Houkin K, Akiyama Y, Wanibuchi M, Mikuni N (2015). Predictive factors for epilepsy in moyamoya disease. J Stroke Cerebrovasc Dis.

[11] Suzuki J, Takaku A (1969). Cerebrovascular "moyamoya" disease. Disease showing abnormal net-like vessels in base of brain. Arch Neurol.

